# Expressed Symptoms and Attitudes Toward Using Twitter for Health Care Engagement Among Patients With Lupus on Social Media: Protocol for a Mixed Methods Study

**DOI:** 10.2196/15716

**Published:** 2021-05-06

**Authors:** Alden Bunyan, Swamy Venuturupalli, Katja Reuter

**Affiliations:** 1 Graduate School in Biomedical Sciences Cedars-Sinai Medical Center Los Angeles, CA United States; 2 Division of Rheumatology Cedars Sinai Medical Center Los Angeles, CA United States; 3 Department of Public Health and Preventive Medicine SUNY Upstate Medical University Syracuse, NY United States; 4 Southern California Clinical and Translational Science Institute Keck School of Medicine of USC University of Southern California Los Angeles, CA United States

**Keywords:** health promotion, infodemiology, infoveillance, Internet, listening, lupus, systematic lupus erythematosus, surveillance, Twitter, survey, social media, social network

## Abstract

**Background:**

Lupus is a complex autoimmune disease that is difficult to diagnose and treat. It is estimated that at least 5 million Americans have lupus, with more than 16,000 new cases of lupus being reported annually in the United States. Social media provides a platform for patients to find rheumatologists and peers and build awareness of the condition. Researchers have suggested that the social network Twitter may serve as a rich avenue for exploring how patients communicate about their health issues. However, there is a lack of research about the characteristics of lupus patients on Twitter and their attitudes toward using Twitter for engaging them with their health care.

**Objective:**

This study has two objectives: (1) to conduct a content analysis of Twitter data published by users (in English) in the United States between September 1, 2017 and October 31, 2018 to identify patients who publicly discuss their lupus condition and to assess their expressed health themes and (2) to conduct a cross-sectional survey among these lupus patients on Twitter to study their attitudes toward using Twitter for engaging them with their health care.

**Methods:**

This is a mixed methods study that analyzes retrospective Twitter data and conducts a cross-sectional survey among lupus patients on Twitter. We used Symplur Signals, a health care social media analytics platform, to access the Twitter data and analyze user-generated posts that include keywords related to lupus. We will use descriptive statistics to analyze the data and identify the most prevalent topics in the Twitter content among lupus patients. We will further conduct self-report surveys via Twitter by inviting all identified lupus patients who discuss their lupus condition on Twitter. The goal of the survey is to collect data about the characteristics of lupus patients (eg, gender, race/ethnicity, educational level) and their attitudes toward using Twitter for engaging them with their health care.

**Results:**

This study has been funded by the National Center for Advancing Translational Science through a Clinical and Translational Science Award. The institutional review board at the University of Southern California (HS-19-00048) approved the study. Data extraction and cleaning are complete. We obtained 47,715 Twitter posts containing terms related to “lupus” from users in the United States published in English between September 1, 2017 and October 31, 2018. We included 40,885 posts in the analysis. Data analysis was completed in Fall 2020.

**Conclusions:**

The data obtained in this pilot study will shed light on whether Twitter provides a promising data source for garnering health-related attitudes among lupus patients. The data will also help to determine whether Twitter might serve as a potential outreach platform for raising awareness of lupus among patients and implementing related health education interventions.

**International Registered Report Identifier (IRRID):**

DERR1-10.2196/15716

## Introduction

### Background and Rationale

Lupus is a chronic disease characterized by an autoimmune response that can range in its frequency and affect any part of the body (skin, joints, and organs). It is estimated that at least 5 million Americans have lupus, with more than 16,000 new cases of lupus being reported annually in the United States [1]. The condition strikes mostly women of childbearing age, while women of color are 2-3 times more likely to develop lupus than Caucasian women. However, the disease can present in men and children as well.

Lupus is a difficult disease to diagnose as its symptoms can often mimic those of other diseases [2]. Systemic lupus erythematosus (SLE), the most common form of lupus, has been reported to remain undiagnosed in some populations for an average of 6 years [2]. SLE tends to present more abruptly and cause more damage in patients of color. This often comes in the form of a spike in disease activity called a “flare” and without treatment, can lead to organ damage and failure. Therefore, early diagnosis is essential for patients with lupus [3].

As most people with lupus develop the disease between the ages of 15 years and 44 years, we hypothesize that social media provides a potentially promising tool for raising awareness and supporting early diagnosis and management of lupus. This study aims to shed light on the use of Twitter among patients who publicly discuss their lupus condition on the platform and to assess their attitudes toward using Twitter to engage them with their health care.

### Social Media

The term “social media” describes widely accessible web-based and mobile technologies that allow users to view, create, and share information online and to participate in social networking [4-6]. Social media provides both a unique data source for data mining of health concerns and related attitudes [7,8] and an unprecedented opportunity for delivering information to reach large segments of the population [9] as well as hard-to-reach subpopulations [10,11]. Today, more than 70% of American adults use at least some type of social media [12].

### The Social Network Twitter

The social network Twitter is used by 23% of American adults, and users are diverse, including Hispanics (25%), Blacks (24%), and Whites (21%) [12]. Twitter users can post short messages (tweets) that are limited to 280 characters. They can search for any public message and engage with tweets (ie, they can “like,” reply, and “retweet” [share] them). By default, Twitter account information such as the profile name, description, and location is public unless a user decides to opt out and make an account private [13,14]. Previous research has suggested that Twitter provides a “rich and promising avenue for exploring how patients conceptualize and communicate about their health issues” [15]. The increasing use of Twitter among members of disease communities is further evidenced by the abundance of disease and health topic hashtags used in the messages [16-21]. A hashtag is a word or phrase preceded by a hash or pound sign (#) and used to identify messages on a specific topic (eg, #lupus, #spoonies). However, there is little information about the use of social media among lupus patients as well as their attitudes toward using Twitter for engaging them with their health care [22].

### Previous Research on Social Media and Lupus

The emergence of social media has created new sources of analyzable data [8] and led to new research fields (ie, infodemiology and infoveillance) [7,23]. The data social media users generate through their online activities are referred to as their digital footprint [24] or social mediome [25]. On Twitter, for example, health surveillance researchers have used this data to gain insight into public perspectives on a variety of diseases and health topics such as influenza, autism, schizophrenia, smoking, and HIV/AIDS [26-31]. In some cases, social media user data demonstrated a correlation between disease prevalence and frequency with which Twitter users discussed a disease [32]. The investigators are not aware of lupus-related surveillance research that involved the social network Twitter.

However, previous research examined user-generated content about lupus on Facebook [33]. The authors looked at the representation of health conditions and found that lupus-related pages ranked the highest for patient support [33]. Additionally, a patient commentary highlighted the use of social media, in particular Twitter, among lupus patients to find rheumatologists, specialist care, and peers and to build awareness of their health needs and experiences [34]. To our knowledge, there are no studies that have leveraged Twitter to improve the understanding of attitudes among patients with lupus.

### Study Objective and Research Questions

This study has two objectives: (1) to conduct a content analysis of Twitter data published by users (in English) in the United States between September 1, 2017 and October 31, 2018 to identify patients who publicly discuss their lupus condition and to assess their expressed health themes and (2) to conduct a cross-sectional survey among the lupus patients on Twitter to study their attitudes toward using Twitter for engaging them with their health care.

Our findings will shed light on whether Twitter provides a promising data source for garnering insights and attitudes about lupus expressed among patients. The findings will help to determine whether Twitter might serve as a potential outreach platform for raising awareness of lupus among patients and implementing related health education interventions.

## Methods

This is a mixed methods study that analyzes retrospective Twitter data and conducts a cross-sectional survey among lupus patients on Twitter.

### Data Collection

This study will analyze user-generated posts in English that include keywords related to “lupus” (Multimedia Appendix 1) from the social network Twitter and were published between September 1, 2017 and October 31, 2018. To access public Twitter user data, we used Symplur Signals [35], a health care social media analytics platform. We limited the dataset to posts from users with locations in the United States.

### Search Filters

Twitter posts containing terms related to “lupus” (Multimedia Appendix 1) were obtained for the range between September 1, 2017 and October 31, 2018. We applied the approach suggested by Kim et al [36] to develop the search filters. These terms can appear in the post or in an accompanying hashtag, for example, Lupus or #LupusChat. We selected keywords and hashtags based on expert knowledge (clinicians, social media experts) and used a systematic search of topic-related language based on data in Symplur Signals.

### Data Cleaning and Debiasing

The following types of irrelevant tweets were excluded: (1) non-English language tweets identified using the Liu method [37], (2) retweets (ie, messages shared by Twitter users that other users composed), and (3) messages that originated from outside the United States. Locating users in the United States was accomplished using a mapped location filter provided by Twitter GNIP through the “Profile Geo Enrichment” algorithm (formerly known as GNIP’s Profile Geo 2.0, which was acquired by Twitter) [38]. This Twitter data service is among the most commonly used data sources in academic Twitter surveillance research [39]. To determine a user’s location, the algorithm uses a number of data points including the self-reported “Bio Location” in the Twitter user profile and geotracking data if available. The Profile Geo service adds “structured geodata relevant to the user location value by geocoding and normalizing location strings where possible” [38]. Research using a similar multi-indicator method to infer the location of the user showed the capability of locating 92% of all tweets [40]. However, the Profile Geo service attempts to determine the best choice for the geographic place described in the profile location string. We acknowledge that the results may not be accurate in all cases due to factors such as multiple places with similar names or ambiguous names. If a value is not provided in a user’s profile location field, the Profile Geo service does not provide a classification.

As we attempt to understand attitudes, we relied on machine learning to identify Twitter posts by social bots or marketing-oriented accounts that could possibly influence the results and introduce bias [41,42]. We used the program BotOrNot [43] to identify those Twitter accounts. Messages from these accounts were removed from the dataset to focus on analyzing patient perspective data. The program BotOrNot scores a detection accuracy above 95% [43].

### Data Collection and Confidentiality

#### Database

Study data were collected using the system REDCap (Research Electronic Data Capture) at the University of Southern California (USC). REDCap is a secure, web-based application designed to support data capture for research studies [44]. All analyses will adhere to the terms and conditions, terms of use, and privacy policies of Twitter.

#### Twitter Data

Any identifying and personal health information was redacted from the dataset by the coders. Since the “Tweet ID,” “Tweet URL,” “Profile thumbnail URL,” “Username,” and “Display Name” in the dataset can potentially identify the person directly, we removed these from the initial data collection sheet and used a unique code identifier instead. We maintained the link between the unique code and the identifiable elements in a separate file. We retained the data only for use in this project and destroyed the identifiable (Tweet ID, Tweet URL, Profile thumbnail URL, Username, and Display Name) information prior to the data analysis as requested by the local IRB.

#### Survey Data

The data will be retained in a secure database called REDCap at USC. The anonymous data will be kept for future research. Individuals are informed that they should not participate in the study if they do not want their data kept.

### Survey Study

#### Study Population

The proposed survey study involves contacting lupus patients in the United States who discuss their health in English on Twitter. Eligible survey respondents will be patients with lupus 18 years of age and older. To focus on feasibility, we will limit this pilot to lupus patients who discuss their health on Twitter. Other individuals who talk about how the condition affects a family member or friend (eg, parents, siblings) will be excluded from this study.

#### Survey Development

The goal of the survey is to collect data about the characteristics of lupus patients (eg, gender, race/ethnicity, educational level) and their attitudes toward using Twitter for health care engagement among lupus patients (eg, How concerned are you about researchers using Twitter user information to identify patients with lupus? How interested are you in getting information related to lupus via Twitter? How interested are you in receiving personalized information about ongoing research and clinical research opportunities on Twitter?). The full survey is included in Multimedia Appendix 2.

### Recruiting Patients With Lupus via Twitter

We will conduct self-report surveys via Twitter by inviting all identified lupus patients who discuss their health on Twitter. We will recruit via the project Twitter account using a personalized message package approach (Multimedia Appendix 3) and replying to a user’s most recent Twitter message where they mention their lupus condition. Sending multiple messages will allow us to introduce the research project and research team ensuring investigator transparency, ask recipients to follow the project Twitter account, and remind them of the privacy risks of using Twitter. Via the URL link in the message, interested users will be directed to a webpage (Multimedia Appendix 4) that includes more information about the study [45]. The page will be hosted by the USC Clinical Studies Directory, a public tool that allows anyone to search for clinical research studies at USC. Only those recipients who decide to follow the project account will be able to receive the link to the survey via a private, direct message. The survey will be available in English and can be completed on any computer, tablet, or smartphone. In the case of no response, reminders will be sent up to 4 weeks after the initial contact.

### Consent Procedures

Eligible lupus patients who are 18 years and older will proceed to the information sheet form and access the survey once they give consent via a check box in the survey form.

### Compensation

Survey participants will be able to enter a raffle to win one of three US $100 gift cards after they complete the survey. 

### Data Analysis

#### Coding

We will use a standard coding approach for characterizing the Twitter messages and users. Two independent team members will use a range of text classifiers (Multimedia Appendix 5) to identify a priori and emergent code categories in the Twitter posts. We will further characterize the user of the Twitter accounts who generated the posts (Multimedia Appendix 6) based on information available in a user’s Twitter profile (ie, username, description, profile image). Cohen Kappa will be calculated for each code category to assess interrater reliability [46,47]. Average Cohen Kappa greater than 0.8 for all categories will be considered substantial for this research. The project principal investigators will help to build consensus for instances where coders disagree.

#### Statistical Analysis

The analyses will rely on public, anonymized data, adhered to the terms and conditions, terms of use, and privacy policies of Twitter. This study was performed under IRB approval from the authors’ university. No Twitter posts will be reported verbatim in the report of the findings to protect the privacy of the users. Representative examples of tweets within each category will be selected to illustrate additional themes and will be shown as paraphrased quotes.

We will use descriptive statistics to analyze the data and identify the most prevalent topics in the Twitter content. Units of analysis will be unique terms in posts as well as the number of Twitter messages and users. For each analysis, we will present findings in a confusion matrix where the diagonal line indicates the prevalence of a topic and the off-diagonal lines indicate topic overlap. The number of posts containing 2 or more topics is found at the intersection of the matrix for these topics. We will further describe the patient characteristics such as age, gender, race/ethnicity, and other characteristics and survey responses. We will use multiple regression to assess which variables (eg, demographics) are significantly associated with acceptance of using Twitter for health care engagement. Analyses will be performed in SPSS (v.24), using *P*=.05 for statistical tests.

#### Sample Size Calculation

The sample size estimate (and survey protocol) is based on previous similar research that demonstrated the usefulness of user data to identify and engage cancer patients on Twitter [48].

Twitter data from users in Los Angeles County posted over the course of 12 months were used to identify 134 cancer patients who had discussed their cancer condition on Twitter. Nearly one-quarter (33/134, 24.63%) of them responded positively to the outreach on Twitter that was focused on clinical trial recruitment. As the prevalence of SLE is lower in the United States, with 20-150 reported cases per 100,000 [49], compared to the cancer incidence, which is 439 per 100,000 men and women per year (based on 2011-2015 cases) [50], we anticipate a lower number of people who discuss their lupus condition on Twitter. In this pilot study, we anticipate identifying around 100-300 Twitter accounts of lupus patients across the United States. We expect that at least 25% of these lupus patients, who we will contact to participate in the survey study, will complete the survey.

#### Risk Analysis

This study presents minimal-risk research. We will use public data from the social network Twitter. We will de-identify any subject’s names or Twitter handles, and they will not appear in the analysis dataset. We have implemented a number of measures to ensure data security and confidentiality (see Data Collection and Confidentiality section). We will further abide by USC IRB regulations and the USC Privacy of Personal Information policy. In general, all data will be entered into a computer and database that are password protected. The data will be stored using appropriate, secure computer software and encrypted computers.

### Dissemination of Study Findings

The study authors plan to publish the study findings in a peer-reviewed journal and at topic-related conferences (to be determined at a later date). All listed authors or contributors are compliant with guidelines outlined by the International Committee of Medical Journal Editors for author inclusion in a published work. Furthermore, to support research transparency and reproducibility, we will share the de-identified research data after publication of the study results. We will share the de-identified data on Figshare, a repository where users can make all of their research outputs available in a citable, shareable, and discoverable manner.

## Results

This study was approved by the IRB at USC (Protocol HS-19-00048; Multimedia Appendix 7). Data extraction and cleaning are complete. The detailed data extraction and cleaning flow chart is included in Multimedia Appendix 8. We obtained 47,715 Twitter posts containing terms related to “lupus” from users in the United States published in English between September 1, 2017 and October 31, 2018. After removing duplicates, retweets, non-English tweets, and Twitter posts from commercial and bot-like accounts, 40,885 posts were included in the analysis. Data analysis was completed in Fall 2020.

## Discussion

### Limitations

The generalizability of the study is somewhat limited, and we recognize that the use of social media data could also lead to potential bias. Social media research and social media–based intervention favor those with internet access. Twitter users tend to be younger (38% are 18-29 years of age), college graduates (32%), and located in urban areas (26%) [12]. Nonetheless, it is worth mentioning that social media users have grown more representative of the broader population; for example, they include the Black population (24%) as well as Whites (21%) and Hispanics (25%) [12]. Additionally, Twitter messages from locations outside the United States and messages in other, non-English languages such as Spanish will not be included. It is also possible that fewer lupus patients discuss their health on Twitter than we anticipate. We addressed this issue by searching Twitter data from users across the United States. However, even if we identify lupus patients on Twitter, it is possible that a lower number of them will engage and take the survey. To incentivize survey completion, participants who complete the survey will be able to enter a raffle to win one of 10 US $100 gift cards.

Finally, we will take several steps to reduce the chance of fraudulent survey responses on Twitter, including sharing the survey link only via private messages on Twitter once a user has followed the project account on Twitter. In the case that the majority of users seems reluctant to follow the Twitter account, we will send reminder messages with the personalized link to the survey via a public reply message on Twitter to increase the survey response rate.

### Practical Significance

This pilot project will provide preliminary data and practical insight into the application of publicly available Twitter data to gain a better understanding of lupus patients who publicly discuss their condition on Twitter and their attitudes toward using the platform to engage them with their health care. The data will also help to determine whether Twitter might serve as a potential outreach platform for raising awareness of lupus and implementing related health interventions.

## Appendix

Multimedia Appendix 1 Lupus-related keywords and hashtags used for the Twitter search. The selection is based on data from Symplur Signals.

Multimedia Appendix 2 Survey.

Multimedia Appendix 3 Twitter recruitment messages.

Multimedia Appendix 4 Study information page.

Multimedia Appendix 5 Coding table used for identifying main themes in lupus-related Twitter posts.

Multimedia Appendix 6 Code categories to classify Twitter users.

Multimedia Appendix 7 IRB approval notice.

Multimedia Appendix 8 Data extraction and cleaning flow diagram.

## Abbreviations

IRB: institutional review board
NCATS: National Center for Advancing Translational Sciences
NIH: National Institutes of Health
REDCap: Research Electronic Data Capture
SLE: systemic lupus erythematosus
USC: University of Southern California
