# Supplementary material for: Expressed Symptoms and Attitudes Toward Using Twitter for Health Care Engagement Among Patients With Lupus on Social Media: Protocol for a Mixed Methods Study
Source: JMIR Res Protoc. 2021 May 6;10(5):e15716. doi: 10.2196/15716 (PMC8138711; doi:10.2196/15716)
Supplement: Multimedia Appendix 1 [file resprot_v10i5e15716_app1.pdf]

Multimedia Appendix 1. Lupus-related keywords and hashtags used for the Twitter search. The selection is based on data from Symplur Signals.

Topic	Keyword	Hashtags
**Lupus**	Lupus	#lupus<br>#lupuschat<br>#lupusstyle<br>#WorldLupusDay<br>#TalkToMeAboutLupus<br>#LupusFeelsLike<br>#LupusTaughtMe<br>#SLE
