# Supplementary material for: Expressed Symptoms and Attitudes Toward Using Twitter for Health Care Engagement Among Patients With Lupus on Social Media: Protocol for a Mixed Methods Study
Source: JMIR Res Protoc. 2021 May 6;10(5):e15716. doi: 10.2196/15716 (PMC8138711; doi:10.2196/15716)
Supplement: Multimedia Appendix 2 [file resprot_v10i5e15716_app2.pdf]

Multimedia Appendix 2. Survey

What do you think?

1. I would say that I use Twitter…
    a. Almost never
    b. About once a month
    c. About once a week
    d. Nearly every day

2. Tell us more about why you write about your health and lupus on Twitter
    a. To connect with others who have the disease and may relate to my situation
    b. To spread awareness of the condition
    c. To receive advice from healthcare professionals, e.g., about treatment options
    d. I don’t share much information about my lupus and symptoms on Twitter.
    e. Other, please specify (textbox)

3. How concerned are you about researchers or health professionals using Twitter user information to identify patients with lupus?
    a. Very concerned
    b. Somewhat concerned
    c. Not too concerned
    d. Not concerned at all
    e. Don't know
    f. Feel free to comment and tell us more (textbox)

4. How interested are you in receiving health information related to Lupus via Twitter?
    a. Very interested
    b. Somewhat interested
    c. Not too interested
    d. Not interested at all
    e. Don't know
    f. Feel free to comment and tell us more (textbox)

5. How interested are you in receiving personalized information about ongoing Lupus research and Lupus-related clinical research opportunities on Twitter?
    a. Very interested
    b. Somewhat interested
    c. Not too interested
    d. Not interested at all
    e. Don't know
    f. Feel free to comment and tell us more (textbox)

6. What type of information would you find most helpful to manage your lupus and health? Check all that apply.
    a. Research articles and results
    b. New treatment options
    c. Information on managing Lupus
    d. Information on medications
    e. Information on lifestyle interventions, for example, exercise/diet, tobacco, sun exposure
    f. Information on ongoing and recruiting research studies where you could participate
    g. Health-related technology, for example, apps for wellness, signs and symptoms, and disease tracking
    h. Feel free to comment and tell us more (textbox)

7. How would you like to receive the information on Twitter?
    a. Public replies
    b. Private messages (DM) only
    c. It does not matter
    d. I don't know
    e. Feel free to comment and tell us more (textbox)

<u>Please tell us more about yourself.</u>

8. What is your age? *(Drop-down)*

9. Are you…?
    a. Female
    b. Male
    c. Other
    d. I don't want to share this information.

10. How do you describe yourself? (check all that apply)
    a. African American / Black
    b. American Indian / Alaska Native
    c. Asian / Pacific Islander
    d. Hispanic
    e. Middle Eastern
    f. White
    g. Other
    h. I don't want to share this information.

Thank you for your time! If you want to enter the raffle for one of three $100 gift cards, please share your first name and email. *This information will be stored separately in a secure database, solely for the purpose of the raffle.*

- First name:
- Email:
