# Supplementary material for: Expressed Symptoms and Attitudes Toward Using Twitter for Health Care Engagement Among Patients With Lupus on Social Media: Protocol for a Mixed Methods Study
Source: JMIR Res Protoc. 2021 May 6;10(5):e15716. doi: 10.2196/15716 (PMC8138711; doi:10.2196/15716)
Supplement: Multimedia Appendix 3 [file resprot_v10i5e15716_app3.pdf]

Multimedia Appendix 3. Twitter recruitment messages.

The following messages will be sent to the identified lupus patients as a reply to their latest tweet (Twitter message) where they discuss their health.

Initial Recruitment

Message 1: Dear [Name or Twitter handle]: We noticed your mention of lupus and wanted to connect with you. We’re conducting a research study to better understand lupus patients who use Twitter. We’d like to hear from you, and you get a chance to win a gift card.

Message 2: If you’re interested in participating, please follow this Twitter account so we can send you the link to our 10-question survey via DM. You can find more information about this study and our research team here: [study page URL]

Message 3: Please note: The security of social media is not guaranteed. Contact us about the study. Don’t post if concerned about privacy.

Distribution of survey link via direct (private) message

Message 1: Thank you for following us. Here is the link to our survey: [unique survey URL] After you complete it, you will get a chance to win a gift card. We look forward to hearing from you!
