# Supplementary figures and images for: Expressed Symptoms and Attitudes Toward Using Twitter for Health Care Engagement Among Patients With Lupus on Social Media: Protocol for a Mixed Methods Study
Source: JMIR Res Protoc. 2021 May 6;10(5):e15716. doi: 10.2196/15716 (PMC8138711; doi:10.2196/15716)

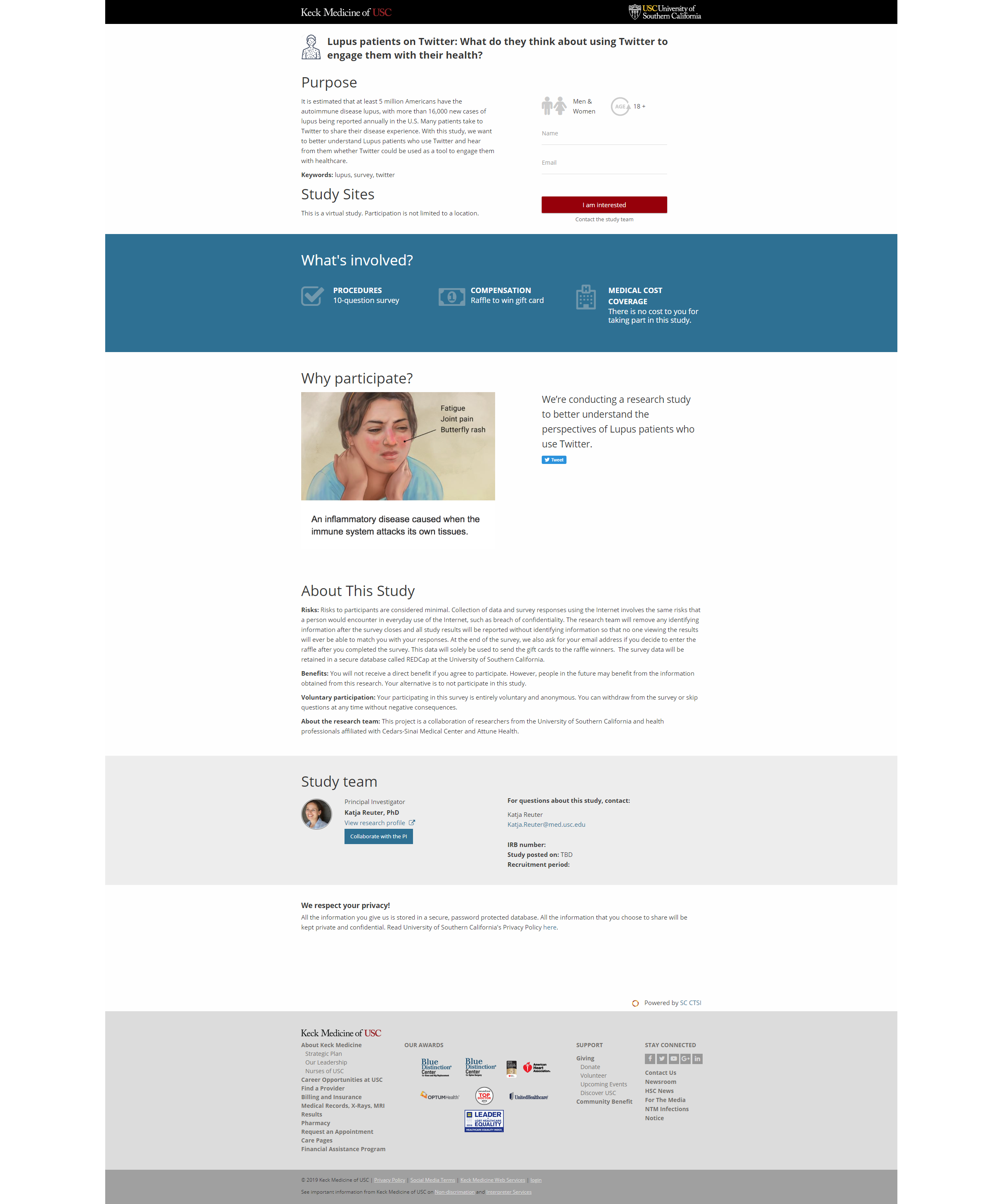

Supplement: Multimedia Appendix 4 [file resprot_v10i5e15716_app4.png]

**Multimedia Appendix 8. Data extraction and cleaning flow diagram.**

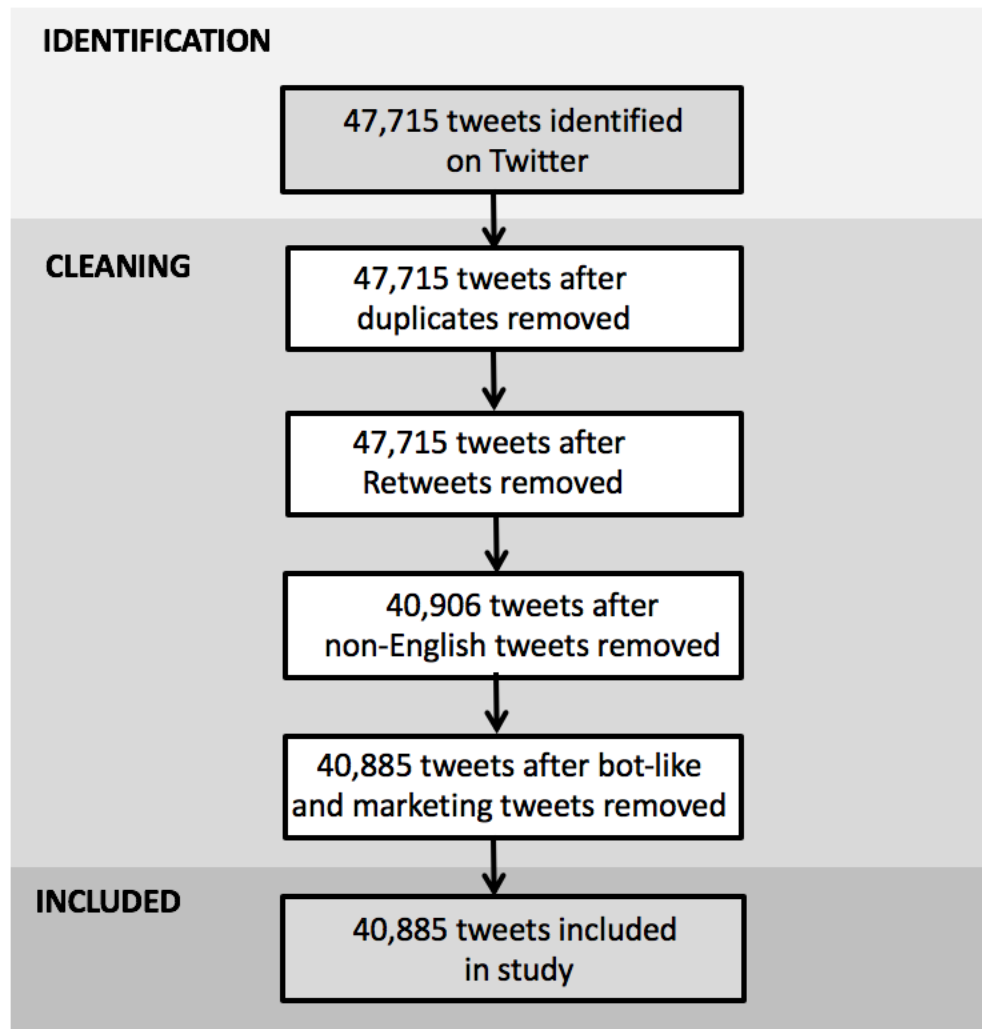

Supplement: Multimedia Appendix 8 [file resprot_v10i5e15716_app8.pdf]
