# Supplementary material for: Expressed Symptoms and Attitudes Toward Using Twitter for Health Care Engagement Among Patients With Lupus on Social Media: Protocol for a Mixed Methods Study
Source: JMIR Res Protoc. 2021 May 6;10(5):e15716. doi: 10.2196/15716 (PMC8138711; doi:10.2196/15716)
Supplement: Multimedia Appendix 5 [file resprot_v10i5e15716_app5.pdf]

Multimedia Appendix 5. Coding table used for identifying main themes in lupus-related Twitter posts.

Code category	A priori codes/variables and definitions (code value)	Emergent codes/variables and definitions (code value)
**Lupus-related symptoms**	Flares (defined as mention of flares and remissions, or a state suggestive of heightened disease activity)<br>Fatigue<br>Rash<br>Butterfly rash<br>Fever<br>Skin sensitivity<br>Headache<br>Mouth sores<br>Nose sores<br>General pain<br>Muscle pain/Myalgia<br>Joint pain/Arthralgia<br>Swollen joints<br>Leg swelling<br>Lymph node enlargement<br>Loss of appetite<br>Digestive issues<br>Pyrexia<br>Weight loss<br>Nausea<br>Vomiting<br>Discoid Lesions<br>Alopecia<br>Panniculitis<br>Edema<br>Skin lesions<br>Swollen fingers	

	Sclerodactyly<br>Calcinosis<br>Telangiectasia/Spider veins<br>Confusion<br>Chorea<br>Myelitis<br>Neuropathy<br>Migraines<br>Arthritis<br>Tendonitis<br>Myositis<br>Dyspnea<br>Cardiac failure<br>Effusion<br>Chest pain<br>Livido reticularis<br>Superficial phlebitis<br>Hypertension<br>High blood pressure<br>Psychosis<br>Organic brain syndrome<br>Visual disturbance<br>Cranial nerve disorder<br>CVA/Stroke<br>Vasculitis<br>Hematuria<br>Proteinuria<br>Pyuria<br>Mucosal ulcers<br>Pleurisy<br>Pleuritis<br>Pericarditis<br>Leukopenia<br>Thrombocytopenia	

	Unable to perform usual activities<br>Limited in fulfilling family responsibilities<br>Can not plan activities or events<br>Anxious<br>Depression<br>Difficulty concentrating<br>Self-conscious<br>Seizures<br>Inflammation	
**Medication (defined as: medication lupus patients should or shouldn’t take, stop medication, safe, compatible, unsafe, teratogens, side-effects)**	**NSAIDs**:<br>Aleve (naproxen sodium)<br>Aspirin<br>Tylenol<br>Ibuprofen<br>Celebrex (celecoxib)<br>Voltaren (diclofenac)<br>**Antimalarials:**<br>Plaquenil (hydroxychloroquine)<br>Quinacrine or Atabrine<br>**Corticosteroids**:<br>Prednisone (deltasone)<br>Methylprednisolone<br>Medrol<br>Cortisone<br>Kenalog<br>**Immunosuppresants:**<br>Imuran (azathioprine)<br>Cellcept (mycophenolate)<br>Trexall, Rasuvo, or Rheumatrex (methotrexate)<br>Arava (leflunomide)<br>Azulfidine (sulfasalazine)<br>Tacrolimus<br>Dapsone<br>Actemra (tocilizumab)<br>Kevzara (sarilumab)<br>**Biologics:**	

	Cimzia (certolizumab pegol)<br>Rituxan (rituximab)<br>Benlysta (belimumab)<br>Enbrel (etanercept)<br>Humira (adalimumab)<br>Remicade (infliximab)<br>Stelara (ustekinumab)<br>Cosentyx (secukinumab)<br>**Anticoagulants:**<br>Heparin<br>Warfarin<br>Coumadin<br>**JAK Inhibitors:**<br>Olumiant (baricitinib)<br>Xeljanz (tofacitinib)<br>**TNF blockers:**<br>Simponi (golimumab)<br>Nerventra (laquinomod)<br><br><br><br><br>**Other treatments:**<br>Acthar (corticotropin)<br>Immune boosters<br>Cytoxan or Neosar (cyclophosphamide)	
