# Supplementary material for: Expressed Symptoms and Attitudes Toward Using Twitter for Health Care Engagement Among Patients With Lupus on Social Media: Protocol for a Mixed Methods Study
Source: JMIR Res Protoc. 2021 May 6;10(5):e15716. doi: 10.2196/15716 (PMC8138711; doi:10.2196/15716)
Supplement: Multimedia Appendix 6 [file resprot_v10i5e15716_app6.pdf]

Multimedia Appendix 6. Code categories to classify Twitter users.

Code category	A priori codes/variables and definitions (data dictionary code value)	Emergent codes/variables and definitions (code value)
**Lupus Status**	• Patient with lupus<br>• Partner of patient with lupus<br>• Healthcare professional<br>• Other or could not be determined	
**Sex**	• Female<br>• Male<br>• Transgender<br>• Could not be determined	
**Race**	• White or Caucasian<br>• Person of color<br>• Could not be determined	
