# Supplementary material for: Expressed Symptoms and Attitudes Toward Using Twitter for Health Care Engagement Among Patients With Lupus on Social Media: Protocol for a Mixed Methods Study
Source: JMIR Res Protoc. 2021 May 6;10(5):e15716. doi: 10.2196/15716 (PMC8138711; doi:10.2196/15716)
Supplement: Multimedia Appendix 7 [file resprot_v10i5e15716_app7.pdf]

iStar

Proposal #HS-19-00048

University of Southern California Institutional Review Board
1640 Marengo Street, Suite 700
Los Angeles, California 90033-9269
Telephone: (323) 442-0114
Fax: (323) 224-8389
Email: irb@usc.edu

Date: Apr 23, 2019, 10:56am

To: Katja Reuter, PhD
Assistant Professor Of Clinical Preventive Medicine
PREVENTIVE MEDICINE

From: University of Southern California Institutional Review Board
1640 Marengo Street, Suite 700
Los Angeles, California 90033-9269
(323) 442-0114

TITLE OF PROPOSAL:
Lupus patients on Twitter: Expressed symptoms and attitudes toward public health promotion interventions on the platform (Lupus patients on Twitter)

Action Date: **4/23/2019** Action Taken: **Approve**

Committee: Institutional Review Board Chairman

Note: The University of Southern California Institutional Review Board (USC IRB) designee determined that your project meets the requirements outlined in 45 CFR 46.110 categories (5) and (7) to receive expedited review. This study was found to involve no more than minimal risk and was **approved on 04/23/2019**. In approving this research, the IRB determined that all of the requirements under 45 CFR 46.111 were satisfied and is not subject to continuing IRB review.

If there are any modifications to the study which changes the risk, scope, or funding status, you are required to submit an amendment to the IRB for review and approval.

The materials submitted and considered for review of this project included:
1. Lupus Social Media Project Study, dated 03-27-2019
2. Transcript data collection form, dated 03-27-2019
3. Information Sheet, dated 04-18-2019
4. Recruitment Twitter message, dated 03-27-2019
5. Study webpage, dated 03-27-2019

You must use the data collection form submitted.

To access IRB-approved documents, click on the "Documents" tab on the main study page.

Minor revisions were made to the recruitment and consent documents by the IRB Analyst (IRBA). See the attached documents for the changes.

WAIVER OF SIGNED CONSENT:
The request for a waiver of signed consent has been approved under 45 CFR 46.117(c)(2).

Principal Investigator Responsibilities:
As the Principal Investigator, you are required to ensure that this research, and the actions of all project personnel involved, will conform with the protocol and its modifications as approved by the IRB; as well as HHS regulations (45 CFR 46); IRB Policies and Procedures; and applicable state laws.

You must inform the IRB immediately if you become aware of any violations to the approved protocol, HHS regulations (45 CFR 46; IRB Policies and Procedures or applicable state laws. You are responsible for reporting any unanticipated adverse events or injuries to the IRB no later than 10 business days from their time of occurrence, using the "Reportable Event" activity in iStar. You are also required to inform the IRB immediately of any significant negative changes in the risk/benefit relationship of the research; as well as of any actions by the sponsor or funding agency, including warnings, suspension or termination of your participation in this research. Failure to comply may result in suspension or termination of the research project, notification of appropriate governmental agencies by the IRB, and/or

suspension of your freedom to present or publish results.

Any proposed changes in the research project must be submitted, reviewed and approved by the IRB before they can be implemented. The only exceptions are changes necessary to eliminate apparent immediate hazards to the research subjects, which can be made immediately, and must be reported to the IRB within 5 business days of implementation. All submissions, including new applications, contingency responses, amendments and continuing reviews are reviewed in the order received.

You must maintain all required research records and recognize that the IRB is authorized to inspect these records at its digression. A final progress report is required by the IRB upon completion or termination of the study.

The principal investigator for this study is responsible for obtaining all necessary approvals before commencing research. Please be sure that you have satisfied applicable requirements, for example: conflicts of interest, bio safety, radiation safety, biorepositories, credentialing, data security, sponsor approval, clinicaltrials.gov or school approval. IRB approval does not convey approval to commence research in the event that other requirements have not been satisfied.

Attachments: IRBA revised Information Sheet_04-18-2019.docx
IRBA revised Study webpage_04-18-2019.docx

Approved Documents: view

This is an auto-generated email. Please do not respond directly to this message using the "reply" address. A response sent in this manner cannot be answered. If you have further questions, please contact your IRB Administrator or IRB/CCI office.

The contents of this email are confidential and intended for the specified recipients only. If you have received this email in error, please notify istar@usc.edu and delete this message.

1. Project Identification and Abstract

1.1. * **Type of Submission**:

- (selected) **Research Protocol or Study on Human Subjects**
- Use of Humanitarian Use Device (Not Research)
- Rely on another IRB (Ceded)

1.2. * **Full Title of Research Protocol**
Lupus patients on Twitter: Expressed symptoms and attitudes toward public health promotion interventions on the platform

1.3. * **Short Title**
Lupus patients on Twitter

1.4. **Abstract: Provide a simple explanation of the study and briefly address (in 1 to 2 sentences) each of the following points: rationale; intervention; objectives or purpose; study population or sample characteristics; study methodology; description of study arms (if appropriate); study endpoints or outcomes; follow-up; statistics and plans for analysis.**
Rationale: Lupus is a chronic disease characterized by an autoimmune response that can range in its frequency and that can affect any part of the body (skin, joints, and/or organs). Some studies have found communication between provider and patient to be a major barrier to diagnosis [ ]. It would be advantageous to investigate other tools that can be used to identify patients expressing symptoms suggestive of the early stages of lupus outside of the clinic.

Objectives: The objective of this study is to conduct a content analysis of Twitter data published by users in the U.S. between 9/1/2017 and 10/31/2018 in order to evaluate if (1) Twitter is a platform in which individuals express symptoms that could be related to lupus, and (2) to learn more about their attitudes toward public health promotion interventions on Twitter. The investigators are not aware of similar that involved the social network Twitter.

Study population: Twitter user who discuss lupus

Methodology: This is a mixed-methods study that includes the analysis of retrospective Twitter user data and a cross-sectional survey.

Analysis: Coding - Text classifiers will be used to identify topics in posts. Two independent team members (the PI and a student) will review the Twitter messages to code them. Statistical analysis - We will use descriptive statistics to analyze the data and identify the most prevalent topics in the Twitter content.

1.5. * **Select which IRB you are requesting review from:**
USC-Health Sciences (HSC)

1.6. * **To the investigator's knowledge, does the Institution have financial and/or intellectual property interests in the sponsor or the products used in this project?**
An institutional conflict may occur when a financial interest of the institution has the potential to bias the outcome of research conducted by its employees or students or to create an unacceptable risk to human subjects.

○ Yes ● No

2. Study Personnel

2.1. Study Personnel and their roles:

	Last Name	First Name	Organization	Study Role	Certifications	Obtain Consent	Interact with Participants	Access Identifiable Data
View	Reuter	Katja	PREVENTIVE MEDICINE	Principal Investigator	HS	yes	yes	yes
View	Bunyan	Alden	USC-Health Sciences (HSC)	Student	HS	no	no	no

<u>Who may be included as "key personnel" on an IRB submission?</u>

Key Personnel are individuals who contribute to the scientific development or execution of a project in a substantive, measurable way, whether or not they receive salaries or compensation under the protocol. Individuals who should be named on an IRB application are those who engage in the following:

a. conducting research through an interaction or intervention with human subjects for research purposes
b. participating in the consent process by leading it or contributing to it
c. directly recording or processing identifiable private information, including protected health information, related to those subjects for the purpose of conducting the research study

<u>Who should NOT be listed as key personnel on an IRB submission:</u>

Individuals paid by the institution to perform a service not part of or paid by the research project performing services that are typically performed for non-research purposes or fee for service:

- honest broker
- pharmacy employees dispensing investigations drugs
- hospital employees obtaining blood through a blood draw or collect urine and provide such specimens to investigators as a service
- radiology clinic employees performing chest x-rays and sending results to investigators as a service
- routine laboratory analyses of blood samples for investigators as a commercial service
- transcription of research study interviews as a commercial service
- not administering any study intervention being tested or evaluated under the protocol

2.2. Is the Principal Investigator a student, resident, fellow, postdoctoral scholar, other trainee, or visiting scholar at USC/CHLA?

○ Yes ● No

2.4. Does this study require Cancer Center Committee (CIC) approval?

○ Yes ● No

2.4.1. Are Cancer Patients Involved? ○ Yes ● No

2.5. Specify the group/organization who has reviewed this study for scientific merit:

○ Federal Agency (e.g. FDA, NIH, CDC, DOE, NSF, DOJ, etc.)

- ○ USC Norris Clinical Investigations Committee
- ○ Doctoral Dissertation Committee
- ○ Other
- ● **None**

3. Required Approvals (for a study already submitted to the IRB)

This screen indicates the approvals received once the proposal has been submitted.

3.1. Pending Division/Department Approvals:

Name	Division/Department	Parent Campus

There are no items to display

3.2. Received Division/Department Approvals:

Name	Division/Department	Parent Campus
PREVENTIVE MEDICINE	Department	USC-Health Sciences (HSC)

3a.3. Other campus committees, services or departments that need to review and approve this protocol:

Committee Name	Committee Chair	Approval Memo

There are no items to display

3a.4. Will the research be conducted through the CTU?

○ Yes ● **No**

4. Funding Information

4.1. * **What existing, planned, or pending support will be used for this study?** (check all that apply)

- ☐ Cooperative Group (SWOG, COG, RTOG, etc.)
- ☑ **CTSI**
- ☐ Department of Defense (DOD) Funds
- ☐ Departmental/Institutional Funds
- ☐ Federal Grant/Contract

- [ ] Foundation Grant/Contract
- [ ] Industry
- [ ] Intramural/Internal Grant
- [ ] Residual Funds
- [ ] State or Local Grant/Contract
- [ ] Subcontract from another institution
- [ ] No Funding
- [ ] Other

4.1.1 Will you be submitting a Just-in-Time (JIT) request?

○ Yes ● **No**

4.2. If the funding source has undergone separate review by the IRB (i.e., cooperative group grants, umbrella grants, multi-project/program grants, center grants), try to select it from the list using the "Add" button. If the funding source is not displayed in the list, enter the information in question 4.4.

Grant #	Principal Investigator	Grant Title
There are no items to display		

4.2.1. If the grants selected in question 4.2 fund multiple studies, please attach the specific pages of the grant that are relevant to THIS study.

Name	Version	Modified
There are no items to display		

4.3. If applicable, select a clinical trial from the TRUE2 system: (Important Note: As of April 1, 2016, the TRUE2 system has been replaced by OnCore for new clinical trial submissions)

4.4. Add the details of each source of funding for this study.

Sponsor	Principal Investigator	Type of Funding
There are no items to display		

4.5. For those studies with a related award in the USC award system, Kuali Coeus, please use the "Find Now" button below to relate this study with the award(s):

Related Awards (uncheck checkbox to remove):

PI First	PI Last	Institutional Proposal	USC Award	Project Title	Prime Sponsor	Sponsor	Project Start	Co

Name	Name	Number	Number		Name	Name	Date	Investigators
Thomas	Buchanan	00080986	006905-00003	Southern California Clinical and Translational Science Institute	US-National Institutes of Health	US-National Center for Advancing Translational Sciences	7/1/2016	

5. Type of Study Review

5.1. Select the type of review that you are requesting for this study:

- ○ Full Committee Review
- ● **Expedited Review**
- ○ Exempt Review
- ○ Coded Specimens/Data

5.2. Attach the protocol here. For simple studies, a separate protocol may not be necessary. However, larger, complex, or multi-site studies require a fully developed protocol. If you have questions contact the IRB office to discuss.

Name	Version	Modified
Lupus Social Media Project_v2_03.27.2019	0.02	3/27/2019 10:15 PM

For investigator-initiated trials at USC, a protocol template and protocol writing tips are available here: https://oprs.usc.edu/hsirb/biomedical/investigator-initiated-trials/

5.3. Attach the sponsor's template informed consent here.

Name	Version	Modified
There are no items to display		

5.4. If any study documents are password protected, enter the passwords here.
N/A

5.5. If there is a sponsor protocol number associated with this file, specify it here:
N/A

6. Study Locations

6.1. Select each campus the study will be associated with (check all that apply):

- ☑ **HSC - Health Sciences Associated Locations**
- ☐ UPC - University Park Associated Locations

☐ CHLA

6.2. Will any research covered by this application be conducted at any other site not affiliated with USC or CHLA?

○ Yes ● **No**

6a. HSC Location(s)

This screen is required if you indicated HSC - Health Sciences Associated Locations (Question 6.1.)

6a.1. Locations that recruitment, consent, and/or study procedures will be performed: (check all that apply)

	Location
☐	LAC+USC Medical Center
☐	LAC+USC Emergency Dept
☐	LAC+USC Outpatient Clinics
☐	LAC+USC 5P21 Building
☐	Keck Hospital of USC Facilities
☐	USC Norris Comprehensive Cancer Center Facilities
☑	**Keck School of Medicine of USC**
☐	USC Center for Health Professions (CHP)
☐	USC School of Dentistry
☐	El Monte Comprehensive Health Center *
☐	H. Claude Hudson Comprehensive Center *
☐	Roybal Comprehensive Health Center *
☐	Verdugo Hills Hospital
☐	Other location (e.g., subjects home, community)

6a.2. Describe other location(s) at HSC:
N/A

6a.3. If you are conducting this research in an LAC+USC location, specify the room numbers:
N/A

6a.4. If you are conducting this research at a location marked with an asterisk "*", attach a letter of approval from the medical director.

Name	Version	Modified
There are no items to display		

9. Methods and Procedures - Selected Descriptors/Community Engaged Research

Note: The list of items below IS NOT an all-inclusive list of methods and procedures available to investigators. The list only includes items that will trigger additional questions specific to areas of research or are necessary for the review process.

9.1. This study will involve: (check all that apply)

- [x] Prospective collection of data/specimens
- [x] Use of existing or retrospective data/specimens

9.2. Study Procedures: (check all that apply)

- [ ] Audio/Video Recordings or Photographs
- [ ] Behavioral Observations and/or Behavioral Experimentation
- [ ] Behavioral Interventions
- [ ] Deception
- [ ] Interview/Focus Groups
- [ ] Population-based Field Study
- [ ] Psychophysiological Testing
- [x] Surveys/Questionnaires/Psychometric Testing
- [ ] Anatomic Pathology Specimens
- [ ] Approved/Investigational Devices
- [ ] Approved/Investigational Drugs and Biologics
- [ ] Biohazardous Substances (e.g. fresh tissue or tissue fluids, infectious agents, microorganisms, recombinant DNA, or shipment of biological material)
- [ ] Blood Collection
- [ ] Controlled Substances
- [ ] Creation of a Data or Tissue Repository

☐ Emergency Research (with exception from informed consent requirements)

☐ Gene Transfer Study

☐ Heritable Genetic Specimens or Germ Line

☐ Magnetic Resonance Imaging (MRI) or ultrasound other than clinically indicated

☐ Radiation Exposure Other Than Clinically Indicated Tests and/or Therapy (e.g. x-ray, CT, DEXA, radiation therapy, etc.)

☐ Stem Cell Research

☐ Substance Abuse Treatment (with medication)

☐ Other Medical Procedures/Considerations

9.3. Is this a clinical trial? [The NIH defines a clinical trial as a prospective research study to evaluate the effects of one or more interventions on health-related biomedical or behavioral outcomes.]

○ Yes ● **No**

9.6. Does your study involve community-engaged research (community-engaged research addresses community needs and involves the community in research plan, conduct of study, etc)?

○ Yes ● **No**

10. Characteristics of the Study Subject Population

10.1. What is the maximum number of subjects you plan to recruit for this site? (Integer values only)

500

10.1.1. If this is a multi-site study, indicate the projected total subject accrual. (Integer values only)

10.1.2. If necessary, provide further explanation of accrual goals for all subject populations.

We plan to recruit all lupus patients we identify on Twitter.

10.2. Describe the inclusion criteria for enrollment. **(HSC: *Refer to specific sections of the protocol/grant, if applicable*)**

Eligible survey respondents will be patients with lupus 18 years of age and older. To focus on feasibility, we will limit this pilot to lupus patients who discuss their health on Twitter. Other individuals who talk about how the condition affects a family member or friend (e.g., parents, siblings) will be excluded from this study. Please see section 2.4 in the study protocol.

10.3. Describe the exclusion criteria for enrollment. **(HSC: *Refer to specific sections of the protocol/grant, if applicable*)**

Lupus patients younger than 18 will be excluded from this pilot study. Please see section 2.4 in the

study protocol.

10.3.1. If there are any age, ethnic, language, or gender-based exclusion criteria, please provide justification.
N/A

11. Research Objectives and Background

11.1. Describe the specific objectives or aims of the study and hypotheses or research questions. (HSC: *refer to specific sections of the protocol/grant, if applicable*)
The objective of this study is to conduct a content analysis of Twitter data published by users in the U.S. between 9/1/2017 and 10/31/2018 in order to evaluate if (1) Twitter is a platform in which individuals express symptoms that could be related to lupus, and (2) to learn more about their attitudes toward public health promotion interventions on Twitter. The investigators are not aware of similar that involved the social network Twitter. Please see section 1.5 in the study protocol.

11.2. Provide a summary of the background of the study, and explain how this research will contribute to existing knowledge. Describe previous work that provides a basis to show that the proposed research can be carried out without undue risk to human subjects. Include relevant citations. (HSC: *refer to specific sections of the protocol/grant, if applicable*)
Our findings will shed light on whether Twitter provides a promising data source for garnering insights and attitudes about lupus expressed among patients and healthcare providers. The data will also help to determine whether Twitter might serve as a potential outreach platform for raising awareness of lupus among patients and healthcare providers and implementing related health interventions. Please see sections 1.1- 1.4 in the study protocol.

12. Methods and Procedures - Prospective Studies

12.1. Describe in detail the design and methodology of the study. Provide a detailed description of the planned data collection, specific outcomes, and criteria for evaluation and endpoint definition. If applicable, include information on stratification or randomization plans. Include the frequency and duration of each activity and the total length of subject participation. Identify and distinguish between those procedures that are standard of care and those that are experimental. *(Refer to specific sections of the protocol/grant, if applicable. Describe any differences between the protocol and the local site.)*
This is a mixed-methods study that analyzes retrospective Twitter data and conducts a cross-sectional survey among Lupus patients on Twitter.Please see the attached protocol, section 2 (methods) for more detail.

12.2. Describe the statistical considerations for the study, how the sample size was determined, and how the results will be analyzed, if applicable. (*Refer to specific sections of the protocol/grant, if applicable*)
Coding: Text classifiers (Table 2) will be used to identify topics in posts. Posts will be classified manually into the a-priori and emergent specific categories. Two independent team members will review the Twitter messages to code them based on the coding criterions. Please note that the coders will remove any identifiable information from the Twitter message trasncripts in the dataset that they might detect during coding.

Statistics: We will use descriptive statistics to analyze the data and identify the most prevalent topics in the Twitter content. Units of analysis will be unique terms in posts as well as the number of Twitter messages and users. We will also describe the patient characteristics such as age, gender, race/ethnicity, and other characteristics and survey responses. We will use multiple regression to assess which variables (e.g., demographics) are significantly associated with acceptance of using Twitter for healthcare engagement. Analyses will be performed in SPSS (v.24); a = 0.05 for statistical tests. Sample size calculation: This is a pilot study and focus will be on describing the

size of the effect.

13. Methods and Procedures - Retrospective Studies/Existing Data

This screen is required if you indicated the use of existing/retrospective data or specimens (Question 9.1.)

13.1. Do the retrospective/existing data involve records/specimens from deceased individuals?

○ Yes ● **No**

13.2. * **Attach a copy of the Data Collection forms you intend to use. Data Collection forms include a summary of the variables to be recorded from the original source.**

	Name	Version	Modified
	Transcript data collection form_v2_03.27.2019	0.02	3/27/2019 8:26 PM

21. Methods and Procedures - Surveys/Questionnaires/Psychometric Testing

This screen is required if you indicated the use of Surveys, Questionnaires, or Psychometric Testing (Question 9.2.)

21.2. Attach copies of all measures/instruments that will be used for this study.

Name	Version	Modified

There are no items to display

22. Special Subject Populations

22.1. Indicate any special subject populations you intend or expect to enroll in the research:
(check all that apply)

- ☐ Normal Volunteers
- ☐ Employees or Students
- ☐ Adults not Competent to Consent (or likely to lose the capacity to consent during the study)
- ☐ Non-English Speaking Populations
- ☐ Minors (subjects under 18 years of age)
- ☐ Pregnant Women / Human Fetuses
- ☐ Neonates (infants under 30 days old)
- ☐ Prisoners/Detainees

- [ ] Wards
- [x] **None of the above**

23. Study Resources

23.1. Describe the time the investigators have available to conduct and complete the research and justify that it is sufficient. Please check-off the items that apply to this study.

- [ ] Employed interns, residents, fellows, or postdocs with dedicated time to conduct this research.
- [x] **Employed faculty and or staff with dedicated time to conduct this research.**
- [x] **Students with dedicated time as part of their training to conduct this research.**
- [ ] Volunteers
- [ ] Other

23.2. Describe the staff and justify their qualifications. Please check-off the items that apply to this study.

- [ ] All biomedical investigators are privileged and credentialed to perform the study activities in the study locations.
- [x] **All study staff are trained and credentialed to perform the duties assigned to them.**
- [x] **All study staff have fulfilled the training mandated by their respective departments or institutions.**
- [ ] Other

24. Subject Recruitment and Informed Consent

24.1. Recruitment Tools that will be used by the local site (check a box only if your site will control the use or distribution of the recruitment tool): (check ALL that apply)

- [ ] Brochures
- [ ] Clinical Data Warehouse (DEWARS)
- [ ] Email/Electronic Mailing Lists
- [ ] Flyers
- [ ] Letters

- [ ] Newspaper/Magazine Advertisements
- [ ] Radio/Television Announcements
- [ ] Subject or Participant Pools
- [ ] Telephone Scripts
- [ ] Verbal (Personal Solicitation)
- [x] **Websites / Social Media Outlets**
- [ ] Other
- [ ] None of the above

24.1.1. Please specify:
We will contact lupus patients we identify on the social network Twitter based on their conversations about the disease.

24.2. Attach copies of all recruitment tools that will be used by the local site.*(Do not attach any advertising or recruitment materials that will not be used at the local site or under control of the local site.)*

Name	Version	Modified
IRBA revised Recruitment Twitter messages_v2_03-27-2019.docx	0.03	3/27/2019 9:25 PM
IRBA revised Study webpage_04-18-2019.docx	0.04	4/23/2019 10:26 AM

24.3. Informed Consent and Waivers:

**** Please note that child assent and parental permission will be addressed on subsequent pages. Do not complete the following consent questions if adults will not be participating in the study. ****

Check the type(s) of consent or waiver of consent planned for this study: (check ALL that apply)

- [ ] Written/signed consent (participants will sign an informed consent document)
- [x] **An information sheet will be provided and/or verbal consent obtained**
- [ ] Waiver of consent (participants will not be asked to sign a consent document or be given an information sheet)

- [ ] Alteration of the elements of consent (participants will sign a consent document, but one or more of the basic required elements of consent will be altered or waived)

24.4. Select the applicable justification for not obtaining written/signed informed consent:

- (●) **The research is no more than minimal risk of harm to subjects and does not involve any procedures for which written consent is normally required outside the research setting (for example, written consent is not needed for minimal risk surveys or non-invasive health measurements in everyday life).**
- ( ) The only record linking the participant and the research data would be the signed consent document, and the main risk to participants would be a breach of confidentiality (participants could suffer from social stigma or embarrassment or other harms if it became known that they participated in research that identified them as having issues including, but not limited to, risky sexual behaviors, drug use, HIV or mental health problems). NOTE: THIS STILL REQUIRES SUBMISSION OF CONSENT FORMS AND DOCUMENTATION OF THE SUBJECTS' WISHES.
- ( ) The subjects or legally authorized representatives are members of a distinct cultural group or community in which signing forms is not the norm, that the research presents no more than minimal risk of harm to subjects, and provided there is an appropriate alternative mechanism for documenting that informed consent was obtained.

24.7. Attach copies of the informed consent documents(s), information sheets, and any statements of new information/findings or consent addenda (as applicable) that will be used in this study. This set should also contain any assent or parental permission documents that will be used.

Name	Version	Modified
IRBA revised Information Sheet_04-18-2019.docx	0.05	4/18/2019 2:52 PM

Click here to obtain an IRB Informed Consent Template and instructions for preparing the consent form.
Consent forms submitted to the IRB should comply with these instructions.

Personnel from section 2.1 obtaining consent/permission/assent:

Last Name	First Name	Organization	Study Role	Certifications	Obtain Consent
Reuter	Katja	PREVENTIVE MEDICINE	Principal Investigator		yes

If the above list is incomplete or incorrect, please navigate to item 2.1 and make your changes there.

24.8. Describe the circumstances and location of the process of informed consent: (check ALL that apply)

- [ ] In a private area
- [ ] In a waiting room, open ward, group, or public setting
- [ ] Online, over the telephone, by mail, or via fax
- [x] **Other**

24.8.1. **If Other, please specify:**
An information sheet will be provided and consent obtained via an electronic form (check box) in a secure database and survey system: USC's REDCap. No signature will be required.

24.9. **Describe how you will assess the individual's comprehension of the research and what it means to participate, including understanding of the voluntary nature of participating.** (check ALL that apply)

- [ ] An assessment tool will be used. (attach a copy of the tool below)
- [ ] This will be verbally assessed. Individuals will be asked to answer the following questions (as applicable): (1) What are you being asked to do? (2) What question is this study trying to answer? (3) What are the potential risks of participating in this study? (4) How often will you need to come in for study visits? (5) What is the difference between participating in this study and your standard medical care? (6) What should you do if you decide to withdraw from the study?
- [x] **Other (specify below)**

24.9.2. **If Other, please explain:**
The information sheet we provide explains the study details. The sheet clearly states that participation is voluntary. The information sheet will include a statement that participants have read the consent, attest that they are 18 years old or older and voluntarily agree to participate before they can proceed with the survey.

24.10. **Describe all measures that will be taken during the recruitment and consent process to ensure that individuals have adequate time to consider participation and to safeguard against potential coercion and undue influence:** (check ALL that apply)

- [x] **The informed consent process will begin with a concise and focused presentation of the key information that is most likely to assist a prospective participant in understanding the reasons why one might or might not want to participate in the research**
- [x] **They will not be forced, threatened or coerced in any way to participate in this research, and no undue influence or other form of constraint will be used to recruit individuals to participate in this research or to retain currently enrolled subjects. (Note: "Coercion" is the use or threat of the use of force to gain compliance. "Undue influence" is when an individual who is in a position of authority (e.g., physician, teacher, employer) exerts inappropriate or excessive manipulation to gain power and compliance over a vulnerable individual (potential research subject). "Constraint" means force, obligation, or pressure.)**
- [ ] They will not be punished or denied something which they would normally receive (e.g., threatening to withdraw health services to which an individual would otherwise be entitled) if they choose not to participate in this research or choose to withdraw early from participation.
- [x] **They will be given an adequate amount of time to consider participation in the study relative to the initiation of study procedures.**
- [x] **The information presented to individuals during recruitment and consent will reflect that provided in the informed consent document/informed consent script.**
- [ ] The recruitment and consent process will not promise them a certainty of cure or benefit beyond what is outlined in the informed consent document/informed consent script.
- [ ] The recruitment and consent process will take place in an area in which it is possible to maintain privacy and confidentiality.
- [ ] They will be given the opportunity to take the informed consent document home in order to

discuss participation with their family, friends and/or others before making a definitive decision.

- [ ] They will receive payment for their participation, but the amount of payment will be commensurate with their participation (not an inducement for participation), and receipt of the payment will not be contingent upon the individual's completion of the study. (Note: The specific method, schedule, and amount of payment must be outlined in the payment section of the application.)
- [x] **Other (explain below)**

24.10.1. If Other, please explain:
The information sheet we provide explains the study details. Potential study participants can take as much time as they need to read the form and take the survey when they feel ready. We also provide contact information so potential study participants can contact us with questions.

25. Financial Obligation and Compensation

25.1. Financial Obligation: Choose the response that best describes the cost to participants.

- ○ All costs are covered by the sponsor or funder.
- ○ Research costs are paid by the sponsor or funding agency; routine health care costs are the responsibility of the participants and/or their healthcare plans.
- ○ All costs are the responsibility of the participants and/or their healthcare plans.
- ○ Drug trials sponsored by the National Cancer Institute or other national institutes.
- ● **There are no costs related to participation.**
- ○ Other

25.1.A. Consent Text: The following financial obligation statement must be contained in the informed consents for this study: (edit only as necessary. If your study has a contract, this language must be consistent with the contract language)
There is no cost to you for taking part in this study.

25.2. Payment for Participation: Describe how much, if any, financial or other form of compensation will be provided to the subject/family. Describe the requisite conditions that must be fulfilled to receive full or partial compensation. Describe the proposed method of timing and disbursement. If children are involved, please specifically address how the compensation will be distributed to children.
Participants who complete the survey will be able to enter a raffle to win one of three $100 gift cards.

25.3. Research-Related Injury and Compensation for Injury: For studies of greater than minimal risk, if participants require care, medical services, or psychological services as a consequence of the research, who will provide this care? If applicable, describe who will pay for research-related injuries.

Medical and/or psychological care/treatment will be offered. In addition:

- ○ Costs for medical care from research-related injuries will be paid by the sponsor or funder.

- ○ Costs for medical care from research-related injuries will not be paid by the sponsor or funder.
- ○ Study has no sponsor or funder who accepts liability for injury.
- ○ Study funder provides the investigational drug or device, but only accepts liability when instructions followed.
- ○ Other

26. Participant Privacy and Data Confidentiality

26.1. **Privacy Protections:** Privacy is a participant's ability to control how other people see, touch, or obtain information about his/her self. Violations of privacy can involve circumstances such as being photographed or videotaped without consent, being asked personal questions in a public setting, being seen without clothing, being observed while conducting personal behavior, or disclosing information about abortions, HIV status, or illegal drug use.

Select the provisions to protect the privacy of the individual during screening, consenting, and conduct of the research: (check ALL that apply)

- [ ] Research procedures will be conducted in person in a private setting.
- [x] **Data will be captured and reviewed in a private setting.**
- [x] **Only authorized research study personnel will be present during research related activities.**
- [x] **The collection of information about participants is limited to the amount necessary to achieve aims of the research.**
- [ ] Participants will not be approached in a setting or location that may constitute an invasion of privacy or could potentially stigmatize them.
- [ ] Other (specify below)

26.2. **Confidentiality Precautions:** Confidentiality is an extension of the concept of privacy; it refers to the participant's understanding of, and agreement to, the ways identifiable information will be collected, stored, and shared. Identifiable information can be printed information, electronic information, or visual information such as photographs.

How will the research data/specimens be labeled? (check ALL that apply)

- [ ] Data and/or specimens will be directly labeled with personal identifying information. (Identifiable)
- [x] **Data and/or specimens will be labeled with a code that the research team can link to personal identifying information. (Coded)**
- [x] **Data and/or specimens will not be labeled with any personal identifying information, nor with a code that the research team can link to personal identifying information. (Anonymous)**

- [ ] Other (explain below)

26.2.2. If you are recording data in more than one way, please explain and provide justification:

Twitter data: Any identifying and personal health information will be redacted from the dataset by the coders. Since the �Tweet ID�, �Tweet URL�, �Profile thumbnail URL�, �Username� and �Display Name� in the dataset can potentially identify the person directly, we will remove these from the initial data collection sheet and use a unique code identifier instead. We will maintain the link between the unique code and the identifiable elements in a separate file. We will retain the data only for use in this project and destroy the identifiable (Tweet ID, Tweet URL, Profile thumbnail URL, Username and Display Name) information prior to the data analysis.

Survey data: The data will be retained in a secure database called REDCap at the University of Southern California. The anonymous data will be kept for future research.

26.3. Study data/specimen will be stored:

- [ ] Physically
- [x] **Electronically**

Which devices will have study data:

- [x] **Local computers/laptops**
- [ ] Removable drives (USB, external drives)
- [x] **Local Server(s)**
- [ ] External Servers (including cloud based services)

Please confirm that, at a minimum, the following measures will be taken and enforced:

- [x] **Electronic data will be stored with appropriate electronic safeguards, such as unique usernames/passwords, and limited to authorized study personnel. Dual factor authentication will be used, if feasible.**
- [x] **Security software (firewall, antivirus, anti-intrusion) will be installed and regularly updated in all servers, workstations, laptops, and other devices used in the study**
- [x] **All computers with access to study data will be scanned regularly (for viruses and spyware, etc.) and problems will be resolved**

26.4. Will identified data and/or specimens be released to a third party (such as a study sponsor, federal agency, or another institution)?

○ Yes ● **No**

26.5. What will happen to the research data and/or specimens at the conclusion of the study? (check ALL that apply)

- [x] **Direct identifiers and/or the key to the codes will be destroyed upon completion of the research (all data/specimens will be stripped of identifying information and/or the key to codes destroyed, paper documents shredded, electronic files purged, electronic media securely erased)**

- [ ] Retained for study record keeping purposes per institutional policy
- [x] **Retained by the investigator for future research use**
- [ ] Retained for future research use (create data or tissue repository/bank)
- [ ] Restricted use data will be destroyed or returned to the source
- [ ] No direct or indirect identifiers are being collected. The anonymous data and/or specimens will be retained at the discretion of the investigator
- [ ] This research is a clinical trial conducted under FDA regulations. Direct identifiers and/or the key to the codes will be destroyed as directed by the sponsor (IND/IDE holder) in accordance with FDA regulations
- [ ] The NIH requires that the records be retained for three years following the completion of the study
- [x] **Other (specify below)**

26.5.1 Please specify:
Twitter data: Any identifying and personal health information will be redacted from the dataset by the coders. Since the �Tweet ID�, �Tweet URL�, �Profile thumbnail URL�, �Username� and �Display Name� in the dataset can potentially identify the person directly, we will remove these from the initial data collection sheet and use a unique code identifier instead. We will maintain the link between the unique code and the identifiable elements in a separate file. We will retain the data only for use in this project and destroy the identifiable (Tweet ID, Tweet URL, Profile thumbnail URL, Username and Display Name) information prior to the data analysis.

Survey data: The data will be retained in a secure database called REDCap at the University of Southern California. The anonymous data will be kept for future research.

26.6. Do you have, or plan to apply for, a Certificate of Confidentiality for this study?

NOTE: All NIH-funded research that meets the definition of human subjects research (including exempt research in which subjects can be identified), is collecting or using human biospecimens that are identifiable or that have a risk of being identifiable, involves the generation of individual level human genomic data, OR involves any other information that might identify a person is automatically issued a Certificate of Confidentiality by NIH.

○ Yes ● **No**

27. Risk/Benefit Assessment - Risks

27.1. Risks, Discomforts and Potential Harms: Describe the risks associated with each research intervention. Include consideration of physical, psychological, social, and other factors. (check all that apply)

- [ ] Discrimination based on genetic findings.
- [ ] Some people may find it upsetting to learn that they have certain mutations or errors in genes that could lead to future health problems for themselves or their children.

- [ ] Some of the questions may make the participant feel uneasy or embarrassed.
- [x] **There is a small risk that people who are not connected with this study will learn a participant�s identity or their personal information.**
- [ ] The participants are providing highly sensitive, personal information in this study. If people not connected with the study learn this information, they could have problems getting a new job, keeping their current job, finding housing, or getting insurance (health, disability, or life insurance). In highly unlikely situations, they could be charged with a crime.
- [ ] Biomedical risks, including drug, device, biologics, radiation, surgery or other research procedures (please specify).
- [ ] The research includes the risk or disclosure that a participant may engage in self-harm or attempt suicide.
- [ ] Venipuncture risks including: mild discomfort (or pain), bruising and swelling around the puncture site, dizziness or fainting, or infection (rare).
- [ ] Other (specify below)

27.1.1. Describe the biomedical or other possible risks and discomforts participants could experience during this study: **(HSC:** ***refer to specific sections of the protocol, grant, investigator's brochure or product labeling, if applicable*****)**
None

27.2. Describe the precautions that will be taken to minimize risks/harms. (check all that apply)

- [x] **We will use our best efforts to keep the findings in this study as confidential as possible.**
- [x] **Subjects can choose to skip or stop answering any questions that make them uncomfortable.**
- [x] **Data will be coded and identity stored separate from data.**
- [ ] Data will be collected anonymously.
- [ ] Biomedical precautions, including precautions relating to drugs, devices, biologics, radiation, surgery or other research procedures (please specify).
- [ ] Venipuncture by individuals certified and privileged to perform the procedure.
- [ ] Other (specify below)

28. Risk/Benefit Analysis - Potential Benefits and Alternatives

28.1. Describe any potential for direct benefits to participants in the study: (check all that apply)

- [x] **There are no direct benefits to research participants**
- [ ] Improvement in some or all of participants' symptoms
- [ ] Improvement in some or all of participants' survival or longevity

- [ ] Information gained from testing or monitoring procedures
- [ ] Provision of drug or device
- [ ] Reduced side effects
- [ ] Other (explain below)

28.2. Describe potential benefits to society, if any. (check all that apply)

- [x] **The advancement of knowledge**
- [ ] A new treatment or therapy for the condition under study
- [ ] None
- [ ] Other (explain below)

28.3. What are the alternatives to participation? (check all that apply)

- [x] **Not participating**
- [ ] Continue current medical care for their condition
- [ ] Participation in other research studies
- [ ] Palliative care
- [ ] No treatment or therapy
- [ ] Participate in other subject pool activities
- [ ] Other (specify below)

28.4. Risks in relation to benefits:

- [x] **The potential benefits to the research participants justify exposure of the participants to the risks.**
- [ ] The potential benefits to humanity justify exposure of the participants to the risks.
- [ ] Other (specify below)

35. Is the HIPAA Privacy Rule Applicable?

35.1. Do you intend to access, review, collect, use or disclose protected health information (PHI) in your research? Answer yes if you intend to do any of the following:

- Look at medical records (paper or electronic) to identify potential research participants
- Look at clinic logs to identify potential research participants
- Record demographic information obtained from medical records (paper or electronic)

- Record health information obtained from medical records (paper or electronic)
- Obtain information from laboratory reports, pathology reports, radiology reports or images, or other reports from medical or mental health testing and treatment
- Obtain information from medical billing records
- Record or use medical record numbers or other information that could be used to identify an individual (review the list of HIPAA identifiers below)
    - Name/Initials
    - Street address, city*, county*, precinct*, zip code*, or equivalent geocodes*
    - All elements of dates (except year) directly related to an individual (date of birth, admission date, discharge date, date of death)*
    - Elements of date, including year, for persons 90 or older
    - Telephone number
    - Fax number
    - Electronic mail address
    - Social Security Number
    - Medical record number
    - Health plan identification number
    - Account number
    - Certificate/license number
    - Vehicle identifiers and serial numbers, including license plate number
    - Device identifiers and serial number
    - Web addresses (URLs); Internet IP addresses
    - Biometric identifiers, including finger and voice print
    - Full face photographic images and any comparable images
    - Any other unique identifying number, characteristic, or code*

○ Yes ● **No**

39. Conflict of Interest Information

39.1. Indicate the Study team member(s) that have a potential conflict of interest. For each person to be designated, click on his/her name and select the disclosure(s) that should be associated with this study.

Study Staff	Role	Conflicts
Katja Reuter	Principal Investigator	No conflicts identified
Alden Bunyan	Student	No conflicts identified

40. Additional Supporting Documents

40.1. Attach any other documents that have not been specifically requested in previous questions, but are needed for IRB review.

Name	Version	Modified
There are no items to display		

40.2. If there is any additional information that you wish to communicate about the study include it below. Please note, this section should not be used instead of the standard application items.

99. Instructions for Submission

You have reached the end of the application. When you are sure of the content, the following steps may be taken to submit your application for review.

1. Click the **"Finish"** button on the top or bottom application navigator bar to return to the workspace.
2. Use the **Hide/Show Errors** above to determine that all sections of the application are filled out correctly.
3. Use the **"Send Study Ready Notification"** activity to send an email to the Principal Investigator and Co-Investigators with instructions for reviewing and submitting the application.
4. **All listed Co-Investigators (indicated in item 2.1.) must use the "Agree to Participate" activity and answer yes.**
5. Once all the Co-Investigators have agreed to participate, the **Principal Investigator** (indicated in item 2.1.) can submit the application by using the **"Submit Application to ____"**, where ____ indicates the IRB you are submitting to.
6. The PI will have to check the PI endorsement box. The PI will also have to check the student endorsement box if it is applicable.
7. The application is submitted. The state indicator in the top left of the workspace will no longer display Pre Submission.
8. The PI and Study Contact Person will receive an email confirming the application has been submitted.
